# The influence of active coping and perceived stress on health disparities in a multi-ethnic low income sample

**DOI:** 10.1186/1471-2458-8-41

**Published:** 2008-01-29

**Authors:** Jennifer M Watson, Henrietta L Logan, Scott L Tomar

**Affiliations:** 1 Department of Community Dentistry and Behavioral Science, The University of Florida, Gainesville, USA

## Abstract

**Background:**

Extensive research has shown that ethnic health disparities are prevalent and many psychological and social factors influence health disparities. Understanding what factors influence health disparities and how to eliminate health disparities has become a major research objective. The purpose of this study was to examine the impact of coping style, stress, socioeconomic status (SES), and discrimination on health disparities in a large urban multi-ethnic sample.

**Methods:**

Data from 894 participants were collected via telephone interviews. Independent variables included: coping style, SES, sex, perceived stress, and perceived discrimination. Dependent variables included self-rated general and oral health status. Data analysis included multiple linear regression modeling.

**Results:**

Coping style was related to oral health for Blacks (B = .23, p < .05) and for Whites there was a significant interaction (B = -.59, p < .05) between coping style and SES for oral health. For Blacks, active coping was associated with better self-reported health. For Whites, low active coping coupled with low SES was significantly associated with worse oral health. Coping style was not significantly related to general health. Higher perceived stress was a significant correlate of poorer general health for all ethnoracial groups and poorer oral health for Hispanics and Blacks. SES was directly related to general health for Hispanics (.B = .27, p < .05) and Whites (B = .23, p < .05) but this relationship was mediated by perceived stress.

**Conclusion:**

Our results indicate that perceived stress is a critical component in understanding health outcomes for all ethnoracial groups. While SES related significantly to general health for Whites and Hispanics, this relationship was mediated by perceived stress. Active coping was associated only with oral health.

## Background

Extensive research has shown that ethnic/racial health disparities are prevalent in the United States. Mortality and morbidity rates are consistently higher among minority groups than among non-minority groups [1]. These disparities range from the prevalence of specific diseases to access to life saving preventive measures [2]. Understanding what factors influence health disparities and how to eliminate health disparities has become a major research objective [3].

Previous research has shown that self-rated health is strongly and consistently associated with morbidity and is frequently shown to be as good as or better than a physical examination in predicting mortality [4]. Self-rated health is often considered a more comprehensive measure of health than simple rates of mortality and morbidity and health disparities have been clearly demonstrated using a single global measure of health [5].

Oral health has become a critical area in health disparities research as evidenced by the first-ever Surgeon General's Report on Oral Health [6]. Poor oral health causes significant pain, diminishes overall quality of life, and is considered an integral part of general health. For example, recent research findings have pointed to possible associations between chronic oral infections and diabetes, heart and lung diseases, stroke, low birth weight, and premature births [6]. Furthermore, individuals with lower income and members of some racial and ethnic minority groups experience a disproportionate level of oral health problems [6].

Many factors including socioeconomic status (SES), chronic stress levels, discrimination, coping style, ethnoracial group membership, and sex are strongly associated with general and oral health disparities, but the causal mechanisms remain unclear [7,8]. A strong association has been found between chronic stress and poorer health [9-11]. In addition, coping strategies that emphasize actively engaging in problem solving strategies, i.e. "active coping", have frequently been associated with better health outcomes, e.g. [12,13] but studies of health outcomes among Blacks have suggested otherwise [14-17]. The John Henryism Hypothesis (JHH) posits that for Blacks, active coping accompanied by chronic psychosocial stress is associated with elevated risk for negative health outcomes among those without sufficient socio-economic resources [15,18]. For example, studies supporting the JHH typically demonstrate that for Blacks in small, poor, mostly rural communities, high active coping coupled with low SES results in higher hypertension and cardiovascular reactivity [14,15,19,20]. JHH combines psychological and social factors many minorities face to begin to explain health disparities, but the generalizability and the precise parameters for the phenomena are still speculative. However, the JHH has only been applied to samples of African American adults and it is unclear if it would apply similarly to other racial or ethnic groups such as Hispanics or to more general health indicators.

In order to better understand the influence of active coping on health in minorities it is critical that active coping be examined in conjunction with common psychological and social stressors known to be related to health in a large multi-ethnic non-rural sample. It is critical that culturally sensitive measures of active coping, socioeconomic status, and discrimination applicable to both minority and non-minority individuals be used to gain a clear understanding of how these factors interact and relate to health. In addition, a critical and often neglected area – self-rated oral health – should be included as a sensitive marker of both social and biological aspects of health [6].

This study addressed important gaps in the stress and coping literature. Specifically, this study investigated how stress and coping influence not just general health, but oral health as well. In addition, it expanded the JHH hypothesis to a racial/ethic population other than African Americans (Hispanics) to discern whether active coping combined with increased stress was deleterious to general and oral health. The purpose of this study was to assess the impact of active coping on health as well as how the relationship between active coping and health was influenced by common psychological stressors (perceived stress) and social stresses (discrimination, SES) in an urban multi-ethnic sample. Specifically, our hypothesis was that high active coping coupled with low socioeconomic status would predict low self-rated health in all ethnic groups. We hypothesized that perceived stress would relate to general and oral health for all ethnic groups, but perceived discrimination would only relate to general and oral health for minority populations. We also hypothesized that SES would be inversely related to health for all ethnic groups.

## Methods

### Data Source

Data for this study were collected as part of a larger study designed to test the effectiveness of an oral cancer awareness billboard campaign in Florida [21]. Baseline data were collected in Miami-Dade and Duval counties in September and October 2001. A follow-up survey was completed in the same counties in July to September 2002. Miami-Dade County is located in southern Florida and contains 2.3 million residents, 57.3% of whom are Hispanic or Latino in origin and 20.3% who are black or African American. Duval County, which includes the city of Jacksonville, is located in the northeastern part of the state. Duval County contains 792,434 residents, 4.1% of who are of Hispanic or Latino origin and 27.8% of who are black or African American. Data used in this analysis are from the follow-up survey (N = 894). The study was reviewed and approved by the University of Florida Health Science Center Institutional Review Board and followed human subject guidelines for research. Verbal informed consent was obtained from each participant.

### Participants

The sample frame for the survey included households with a land line telephone located within low to moderate income areas within each of the two counties; median household income for the targeted census tracts ranged from $7,243 to $97,453, with a median of $33,252. The inclusion criteria for participation in the study were to be at least 18 years of age and to speak either English or Spanish. Random digit dialing was used to select a probability sample of households. Approximately 25% of the telephone interviewers were Black and 55% were females. Each interview lasted approximately 27 minutes. To complete 894 surveys, 8,863 telephone numbers were selected of which 699 were for businesses, institutions, or group quarters and were ineligible to participate. Because of technical problems (e.g., fax number, answering machines with no message), 3,386 telephone numbers were excluded. There were 132 telephone numbers that reached households in which physical, mental or language barriers made potential respondents ineligible. There were 1,200 persons who refused to participate or did not complete the telephone survey. No eligible respondent was available (including no answer or always busy) at 2,552 telephone numbers (See Table 1). Further details on the survey design and content have been published previously [21].

**Table 1:** Final Phone Call Dispositions.

Category	N
**Completed Interviews**	894
**Eligible No Interview**	
Refusal/less than 50% complete	1200
Non Contact	
**Ineligible Respondents**	
Physical/Mental/language barriers	132
**Unknown Eligibility**	
NO answer/Always busy	2552
**Not Eligible**	
Business/institutions/group quarters	699
Fax/Data line	3386

### Measures

#### Self-rated general health and oral health

Self-rated general health and self-rated oral health were the key dependent variables in this study. Participants were asked to indicate how they rated their health in general and their dental or oral health on a 5-point scale from 1 ("Excellent") to 5 ("Poor"). This method of assessing global general and oral health has been shown to be a valid assessment of health and also has the advantage of permitting respondents to integrate multiple aspects of their health in their rating [4,22-27]. It offers positive response categories (excellent/good) rather then limiting people to only reporting health problems [28]. In order to maintain consistency with large epidemiologic studies that used single global measures of self-rated health, "Excellent" and "Very Good" were combined into a single category [5]. Items were reverse-coded so that higher scores indicated better health.

#### Demographic Information

Demographic items were derived from the Behavioral Risk Factor Surveillance System Survey [29]. Participants indicated their sex (male, female), ethnicity (Hispanic/Latino) and race (White, Black or African-American, Asian, Pacific Islander, American Indian/Native American, Other). For purposes of this analysis, three racial/ethnic groups were used: Hispanics, non-Hispanic blacks, and non-Hispanic whites. Hereafter, non-Hispanic blacks are referred to as Blacks and non-Hispanic whites are referred to as Whites.

#### SES

A modified version of the MacArthur Scale of Subjective Social Status [30] was used to assess the respondents' social status. Subjective social status has been characterized as a person's belief about his or her location in a status order and takes into account multidimensionality of SES simultaneously. It has been shown to assess aspects of social status that are typically not captured by more traditional SES measures such as income or education [31]. Respondents were asked to "think of a ladder with 10 rungs as representing where people stand in the United States. At the top of the ladder (10) are the people who are the best off – those who have the most money, the most education and the most respected jobs. At the bottom (1) are the people who are the worst off – who have the least money, least education, and the least respected jobs or no job..." They were then asked to place themselves on this ladder, at this time in their lives, relative to other people in the United States by selecting a number from 1 to 10. The midpoint of the scale (5) was used to divide participants into low SES vs. high SES.

#### Discrimination

Discrimination was assessed by using "The Everyday Discrimination Scale" [32]. This is a 9 item measure assesses chronic and routine experiences of unfair treatment and has been shown to have reasonable internal consistency (Cronbach's alpha = .88) [32]. It can be easily utilized with all ethnic and racial groups because it does not focus on race or color discrimination solely but rather more typical occurrences of unfair treatment. For instance, participants rate their day-to-day experience of how often they experience unfair treatment; are treated will less courtesy than others. Mean scores were calculated for participants that had valid data on at least 2/3 of the items and higher scores indicate higher discrimination.

#### Stress

Stress was assessed by utilizing Cohen's Perceived Stress Scale (PSS). The PSS is a 14-item scale and is one of the most widely used psychological instruments for measuring the perceptions of stress [33]. Items were designed to assess how unpredictable, uncontrollable and overwhelming respondents find their lives and higher scores indicate higher stress. The PSS was designed for use with community samples with at least a junior high school level of education [33].

#### Active Coping

Active coping was assessed by using the *John Henry Active Coping Scale (JHAC)*. JHAC is a culturally sensitive 12-item scale that assesses a strong personality predisposition to cope actively with psychosocial stressors in one's environment. Participants indicated on a 5-point Likert scale (from 1 = "Completely False" to 5 = "Completely True") how much they agreed with each specific statement (e.g. "I've always felt that I could make of my life pretty much what I wanted to make of it; I am rarely disappointed by the results of my hard work). Higher scores indicate more active coping. No test-retest reliability coefficient of the scale has been published and internal consistency of the scale assessed via Cronbach's alpha range from mid-70s to mid-80s [16,18].

JHAC is scored by summing each participant's responses. If three or fewer items were missing then the average of the non-missing responses was substituted for the missing response. Scores can range from 12 to 60. In keeping with the methodology proposed by James and colleagues, [15,18,34] scores were dichotomized at the median for each racial/ethnic group to categorize respondents into low and high John Henry active coping groups.

### Data Analysis

We first generated univariate statistics to describe demographic characteristic of the sample participants, JH, SES, discrimination, and stress. Pearson correlation coefficients were used to examine the bivariate relationships among active coping, SES, stress, and discrimination. Hierarchical linear regression models were used to analyze the relationship between active coping, stress, sex, SES, and self-rated health. Age was strongly associated with oral and general health, and was therefore included as a covariate in multivariable models.

We performed stratified analysis by race/ethnicity (Black, White, and Hispanic) to determine the association between active coping and self-rated general and oral health. To fully explore the relationship between active coping and self-rated health and to test potential interaction effects we ran a series of linear regression models that examined interaction terms and main effects for active coping, sex, and SES. We examined the additional impact of stress and discrimination on self-rated general health and oral health.

### The John Henryism Hypothesis (JHH)

We ran a series of multiple regression models that examined the impact of JH, SES and sex on self-rated health. First, we ran main effect models. Then to specifically assess the John Henryism Hypothesis we examine two-way interactions between JH and SES and JH and Sex. We then ran a model that examined the 3-way interaction between JH, SES and sex for self-rated health.

### Stress and Discrimination

We conducted analyses to determine the additional impact of discrimination and perceived stress. We first ran a model to examine whether discrimination and stress were significantly related to self-rated health even after accounting for significant effects in the first models (e.g. JH, SES, or sex). We then examined whether JH interacted with either of those constructs. If no interaction terms were statistically significant, we ran a simplified model including only significant effects. This series of modeling was also conducted for self-rated oral health.

## Results

Participants with complete data on all independent variables were included in this analysis (n = 812). Overall, more than one-half of the sample was female, 29% were White, 47% were Black, and 24% were Hispanic. Table 2 provides the distribution of the main independent and dependent variables of interest, stratified by race/ethnicity. Forty-eight percent of the overall sample reported excellent or very good health, and 38% of the participants reported excellent or very good oral health. Black and White participants reported similar levels of oral and general health while Hispanic participants reported the lowest oral and general health ratings. The minimum age in the sample was 18 and the maximum age was 91. Age differed significantly among the ethnic/racial groups and was also significantly correlated with general health (r = -243, p < .001 and oral health (r = -.161, p < .001) and was therefore included as a covariate in all analyses. Cronbach's alpha for the perceived stress scale was .68 and for the discrimination scale was .87 in this sample. There were significant but modest correlations between all of the variables (JH, stress, SES) except the correlation between JH and discrimination was weak and not significant (See Table 3). These findings suggest that although there is some overlap, these variables measure different constructs.

**Table 2:** Selected characteristics of study participants, by race/ethnicity.

Variable	White	Black	Hispanic	P value ^e^
	**(n = 238)**	**(n = 378)**	**(n = 196)**	
	**%**	**%**	**%**	
Sex				
Female	61.7	57.5	54.5	.305
Male	38.3	42.5	45.5	
				
Socioeconomic Status ^a^				
Low (1–5)	42.5	49.9	56.6	.017
High (GT 5)	57.5	50.1	43.4	
				
	**Median**	**Median**	**Median**	
John Henryism (range 12–60) ^b^	52 ^e^	52 ^f^	51 ^e^	.011
				
	**Mean (SD)**	**Mean (SD)**	**Mean (SD)**	
Age	47.53(17.5) ^f^	42.13(15.62) ^e^	41.68(15.27) ^e^	< .001
Self-Rated General Health (range 1–4) ^c^	03.32(00.82) ^e^	03.28(00.85) ^e^	03.04(00.94) ^f^	.001
Self-Rated Oral Health (range 1–4) ^c^	03.13(00.91) ^e^	03.03(00.96) ^e^	02.90(00.98) ^f^	.046
Perceived Discrimination (range 1–6) ^d^	02.18(00.95) ^e^	02.61(01.08)^f^	02.23(01.07) ^e^	< .001
Perceived Stress (3–40) ^d^	22.58(05.87)^e^	22.25(06.20) ^f^	23.91(06.09) ^e^	.010

^a ^Perceived SES in the United states based on respondents rating of how they compare to others in terms of income, education and job status. Higher scores indicate higher SES.

^b ^John Henryism Active Coping assesses predisposition to cope actively with psychosocial stressors in one's environment, higher scores indicated more active coping.

^c ^Based on participants' ratings of their general and oral health, higher scores indicated better health.

^d ^Higher scores indicate higher discrimination or higher perceived stress.

^e ^Analyses were chi square for categorical variables and ANOVA for continuous variables, different subscripts in row indicate a significant difference between groups for ANOVA analyses.

**Table 3:** Correlations^a ^among JH, SES, and Perceived Discrimination (n = 812).

Variable	SES ^b^	Discrimination	Perceived Stress
Discrimination	-.140 *	------	-----
Perceived stress	-.250 *	.206 *	-----
JH	.105 *	-.063	-.227*

^a ^Pearson correlation coefficients.

^b ^SES is a Dichotomous variable (low/high)

* P < .01

### General Health

Table 4 presents parameter estimates from the preliminary models for each ethnic/racial group.

**Table 4:** Preliminary multiple linear regression model parameter estimates for self-rated general and oral health, by race/ethnicity adjusted for age.

**Variables**	**Black**	**Hispanic**	**White**
	**(n = 375)**	**(n = 194)**	**(n = 233)**

**GENERAL HEALTH**	^a ^Beta	Beta	Beta
**John Henryism (JH), Sex, SES**			
JH × Sex × SES	.164	.561	-.012
JH × Sex	.169	-.016	-.411
JH × SES	-.277	.358	-.349
*Main Effects*			
JH	.089	.153	.161
Sex (male)	.152	.019	-.011
SES	.023	.280*	.245 *
**Perceived Stress and Discrimination**			
JH × Perceived Stress	-.006	-.013	-.002
JH × Perceived Discrimination	.046	-.169	-.077
*Main Effects*			
Perceived Stress	-.024 *	-.052**	-.038**
Perceived Discrimination	-.081*	.032	.009
Sex	.163	-.001	-.045
JH	.023	.095	.083
SES	-.062	.115	.131
**ORAL HEALTH**	**(n = 376)**	**(n = 194)**	**(n = 233)**
JH × Sex × SES	.075	.544	.597
JH × Sex	-.024	.112	-.438
JH × SES	-.113	-.074	-.679*
*Main Effects*			
JH	.229*	.068	---
Sex (male)	-.057	.044	-.307**
SES	.111	.229	---
**Perceived Stress and Discrimination**			
JH × Perceived Stress	.003	-.008	.015
JH × Perceived Discrimination	.117	-.048	-.166
*Main Effect*			
Perceived Stress	-.031**	-.038**	-.019
Perceived Discrimination	-. 015	.031	-.070
Sex	-.069	.005	-.301*
JH	.151	.005	.150
SES	.030	.115	.333**

^a ^Unstandardized Coefficients.

^b ^SES is a dichotomous variable (low/high)

* p < .05 ** p < .01

#### Blacks

The JHH was not supported in the Black sample. Sex approached significance (B = .163; p = .052) when accounting for the effects of perceived stress and discrimination. Perceived stress and discrimination were both significantly related to general health. The final model included sex, perceived discrimination and perceived stress. Black males (x = 3.42) reported significantly higher health ratings than did Black females (x = 3.19). For both Black men and Black women, higher stress and higher perceived discrimination was significantly related to worse health (See Table 5 for final models).

**Table 5:** Final multiple linear regression model parameter estimates for self-rated general and oral health, by race/ethnicity adjusted for age.

**Variables**	**Beta ^a^**	**p-value**
***GENERAL HEALTH ***		
**Blacks (n = 375)**		
Sex (Male)	.164	.048
Perceived Stress	-.024	< .001
Perceived Discrimination	-.078	.046
**Hispanics (n = 194)**		
Perceived Stress	-.056	< .001
		
**Whites (n = 233)**		
Perceived Stress	-.042	< .001
		
***ORAL HEALTH***		
		
**Blacks (n = 375)**		
Perceived Stress	-.034	< .001
		
**Hispanics (n = 194)**		
Perceived Stress	-.040	< .001
		
**Whites (n = 233)**		
Sex	.757	< .001
SES ^b ^× John Henryism (JH)		
REF High JH and Low SES	------	
Low JH and Low SES	-.552	.001
Low JH and High SES	.205	.222
High JH and High SES	.077	.631

^a ^Unstandardized Coefficients.

^b ^SES is a dichotomous variable (low/high)

#### Hispanics

The JHH was not supported in the Hispanic sample. The only significant effect was a main effect for SES with low SES participants indicating worse health (x = 2.92) than high SES participants (x = 3.19). When examining the impact of perceived stress and perceived discrimination only perceived stress was significantly related to general health. When perceived stress was included in the model for Hispanics, SES was no longer significantly related to health status. Therefore, the final model included only perceived stress with higher perceived stress being significantly related to worse health.

#### Whites

The JHH was not supported in the White sample. The only significant effect was a main effect for SES, with low SES participants reporting worse health (x = 3.17) than high SES participants (x = 3.43). When examining the impact of stress and discrimination only perceived stress was significantly related to general health. Mirroring the results for our Hispanic sample, when stress was included in the model, SES was no longer significantly related to self-rated health status. Therefore, the final model included only stress with higher stress being significantly related to worse health.

### Oral Health

Table 4 presents parameter estimates from the preliminary models for each ethnic/racial group.

#### Blacks

The JHH was not supported for oral health in the Black sample. However, active coping was significantly related to oral health as a main effect. Black participants with high active coping reported better oral health (x = 3.15) than those with low active coping (x = 2.93). When examining the impact of stress and discrimination only perceived stress was significantly related to oral health. However, when stress was included in the model, active coping was no longer significantly related to self-rated oral health. Therefore, the final model included only stress with higher stress being significantly related to worse oral health. (See Table 5 for final models).

#### Hispanics

The JHH was not supported for oral health in the Hispanic sample. Perceived stress was the only variable significantly related to oral health for Hispanics. Therefore, the final model included only stress with higher stress being significantly related to worse health

#### Whites

When examining the JHH in Whites there was a significant interaction between active coping and SES (B = -.595, p = .011) however, it was participants with low active coping and low SES that reported significantly worse oral health than participants with high active coping and low SES. Sex was the only other variable significantly related to oral health with White males (x = 2.97) reporting significantly lower oral health ratings than White females (x = 3.22). Therefore, the final model included only the significant interaction between active coping and SES and sex.

## Discussion

This study assessed active coping and health, taking into account the influence of known psychological and social correlates of general and oral health in a low-income, ethnically diverse sample. Overall, perceived stress was the strongest correlate of general and oral health and often accounted for a significant amount of the variance associated with SES. Sex of the participant was related to general health only for Black participants and oral health for white participants. The main hypothesis for this study was not supported, as active coping coupled with low SES did not relate to worse self-rated general or oral health in any of the ethnic/racial groups.

Our results suggest that overall perceived stress may be a critical component in understanding health outcomes for low income Black, Hispanic and White adults. Perceived stress was significantly related to reports of general health for all ethnoracial groups and was significantly related to self-rated oral health for Black and Hispanic participants. While SES was significantly associated with general health for Whites and Hispanics, this relationship was moderated by stress. It may be that low SES is a proxy for higher stress because lower SES (lack of economic resources, lower education and lower occupation) is associated with higher stress. These results point to the importance of incorporating measures of stress into health disparities research.

Perceived discrimination was significantly related to general health only for Black participants. Increased discrimination relates to higher stress levels and has been regularly associated with poorer health [9-11]. The fact that discrimination was associated with self-rated health status only among Black participants may be due in part to the location of the survey. A majority of Miami-Dade County's population is comprised of residents of Hispanic or Latino origin (57.3%), and discrimination may be less common in such circumstances.

Sex was significantly related to general health only for Black participants. For Blacks, the relationship between sex and health was not moderated by stress. Research has shown that men and women cope differently in response to stress, and that those differences ultimately can influence physiology and health [35]. Previous researchers have hypothesized that Black women's more diverse roles may be a protective factor for health, while Black men who do not have as pronounced a family role suffer due to economic disparities [16]. Our results indicate that the multiple roles Black women are required to fill may actually be detrimental to their overall health possibly due to the stress of trying to balance numerous critical roles.

It is interesting that coping style was related solely to oral health and not to general health. Although the John Henryism Hypothesis (JHH) was not supported by our data, it is possible that, although the sample size was large, we did not have adequate statistical power to detect such interaction effects. The JHH has generally been linked to conditions that lead to worse health such as high blood pressure or cardiovascular reactivity [14,15]. This relationship makes intuitive sense in that active coping with limited resources can increase activity in the parasympathetic nervous system which could increase heart rate and blood pressure. Our measure of general health was more global than physiological markers such as blood pressure. However, coping did relate to oral health for Blacks and Whites but again not in the way hypothesized by the JHH. There is no reason to expect that active coping with stressors would be independently related to poorer health [12,36]. Our finding that Blacks with active coping strategies reported better health is consistent with an extensive literature on the benefit of active coping to psychological and physical health [12,36]. High active coping may be critical in oral health as several active self-care strategies (e.g. daily tooth brushing and flossing) have been found to dramatically improve oral health. Oral health is a critical component of general health and our results show that key psychological factors (e.g. stress and coping) and social factors (discrimination and SES) impact global measures of oral and general health differently.

The strengths of this study include comprehensive assessment of factors that have been shown to influence health disparities, such as ethnoracial group membership, sex, discrimination, SES and active coping. In addition, this study expanded on past research by examining the JH construct among Hispanics, another U.S. ethnic group that experiences health disparities. This study utilizes an encompassing and culturally relevant measure of SES and discrimination. Finally, health outcomes were assessed utilizing a comprehensive measure of both general and oral health. Oral health is often neglected in disparities research but is an essential factor in overall health [6].

Limitations of this study include reliance on self-reported data, the absence of physiological or clinical measures of oral or general health, and a cross-sectional study design. Because they were not measured, it was also not possible to rule out other unmeasured psychological variables such as neuroticism or depressive symptoms as responsible for the observed association between self rated health indicators and perceived stress. Despite the potential biases inherent in self-rated measures, obtaining objective measures of discrimination and stress would be extremely difficult and the association between self-rated health status and excess mortality has been well-established [4,5,26]. In addition, moving toward a more all-encompassing assessment of health allows for a more comprehensive understanding of health disparities [26]. Finally, although this sample was diverse in regards to ethnicity/race, all participants resided in one of two cities in Florida.

## Conclusion

This study examined several known correlates of health status and factors related to health disparities among a large multi-ethnic sample and also expanded the study of these variables to oral health. Active coping was positively related to oral health only, while perceived stress was an important correlate of both general and oral health. Although SES related to general health for Hispanics and Whites, this relationship was mediated by perceived stress. These results point to the importance measuring perceived stress directly rather than relying on SES as a proxy for stress. Coping styles or perceptions of stress can differ greatly among individuals in identical situations. Simultaneously assessing general health, oral health, and several critical psychological and social variables within a large multi-ethnic sample provides an important step in understanding the complex etiology of health disparities in the United States. Future research should include longitudinal studies to help clarify the temporal relation of these factors.

## Competing interests

The author(s) declare that they have no competing interests.

## Authors' contributions

HL and ST made substantial contributions to the conception, design and acquisition of the data. HL, ST, JW all participated in the data analytic plan and interpretation of data. JW performed all data analysis and initial drafts of the manuscripts. HL, ST and JW all participated in reviewing and revising the manuscript.

## Pre-publication history

The pre-publication history for this paper can be accessed here:


